# Case report: JAKi and TNFi dual therapy is a potential treatment strategy for difficult-to-treat rheumatoid arthritis

**DOI:** 10.3389/fimmu.2022.1074329

**Published:** 2022-12-14

**Authors:** Jing-Wen Chen, Wen-Shuang Zhang, Chang-Song Lin, Qiang Xu

**Affiliations:** ^1^ The First Clinical Medical College, Guangzhou University of Chinese Medicine, Guangzhou, China; ^2^ Department of Rheumatology, The First Affiliated Hospital of Guangzhou University of Chinese Medicine, Guangzhou, China

**Keywords:** rheumatoid arthritis, tofacitinib, adalimumab, JAKi, TNFi, dual therapy

## Abstract

Rheumatoid arthritis (RA) is a heterogeneous chronic disease. RA patients should start disease modifying anti-rheumatic drugs (DMARDs) therapy immediately after diagnosis. If first-line treatment with conventional synthetic DMARDs does not relieve the disease, biology and targeted synthetic DMARDs are options for patients. Patients can switch to different types of biological and targeted synthetic DMARDs if remission is not achieved. However, for patients with difficult-to-treat RA, achieving disease stabilization after the failure of multiple biological and targeted synthetic DMARDs is a clinical challenge that needs to be addressed. As distinct cytokine pathways, the benefits and challenges of dual therapy are worth discussing. As the most extensively used biologic DMARDs, adalimumab is an anti-tumor necrosis factor monoclonal antibody used to treat RA. Tofacitinib, as a Janus Kinase inhibitor, is an orally administered targeted synthetic DMARDs that involved in the regulation of immune responses by directly or indirectly inhibiting cytokine pathways. This report describes a successful case of a 48-year-old woman with difficult-to-treat RA who treated with Tofacitinib combined with adalimumab. She had been on glucocorticosteroid for a long time, but had persistent joint pain and fatigue. At more than one year of follow-up, her Disease Activity Score for 28-joint counts based on the erythrocyte sedimentation rate (DAS28-ESR) remained in complete remission, and she discontinued her glucocorticosteroid medications. Also, she did not develop a mycobacterial tuberculosis infection, herpes zoster, and new-onset cardiovascular events.

## 1 Introduction

Rheumatoid arthritis (RA) is a joint-disabling inflammatory disease associated with synovitis. Patients with RA should start treatment with synthetic modifying anti-rheumatic drugs (DMARDs) as early as possible to prevent or further joint destruction. This disease is challenging to treat in some difficult to treat RA patients using conventional synthetic DMARDs(csDMARDs) and biological and targeted synthetic DMARDs (b/tsDMARDs) (1). Patients with RA can use different b/tsDMARDs if remission is not achieved with their current medication. Even so, special attention should be given to the ability of bDMARDs to trigger latent tuberculosis infection (LTBI) and hepatitis, pneumocystis carinii pneumonia, malignant tumor, etc. (2).

A significant proportion of patients with RA remain symptomatic despite treatment based on current management recommendations; these patients are considered to have ‘difficult-to-treat RA’. Therefore, the following criteria (3) were agreed upon by task force members as mandatory elements of the definition of difficult-to-treat RA: (a) treatment failure history; (b) characterisation of active/symptomatic disease; and (c) clinical perception. These elements were selected based on the results of the survey.

Cytokines regulate multiple inflammatory processes associated with the pathogenesis of RA and are extensively present in the serum and arthritic synovium of RA patients (4). Cytokines such as Interleukin- 6 (IL-6) and tumor necrosis factor (TNF) are key effectors of the tissue response in RA. Stromal elements might act upstream of these cytokines (acting as sentinel cells that recognize danger and damage) and are, therefore, possible drivers of persistent synovitis (5).

Tumor necrosis factor inhibitors (TNFi) were the first category of biologics to emerge. In 2006, Schiff et al. assessed the safety of adalimumab in global clinical trials and post-marketing surveillance of patients with RA. Analyses of these data demonstrated that long-term adalimumab therapy was generally safe and well-tolerated in patients with RA (6). Adalimumab increases the risk of non-serious infections, such as upper respiratory tract infections, sinusitis, flu syndrome, and urinary tract infections (7).

Janus kinase (JAK) and signal transducer and activator of transcription (STAT) signaling in multiple disease has led to an increasing applicability of therapeutic intervention with Janus kinase inhibitor (JAKi). JAKi is critical for the signaling of cytokines that bind to type I and II cytokine receptors, including cytokines responsible for driving inflammatory processes implicated in the pathogenesis of RA (e.g., type I interferons and several interleukins) (8, 9). JAKi have been developed as cytokines therapy. Tofacitinib (JAK-1/3i) and baricitinib (JAK-1/2i), have been approved for the treatment of RA in China. JAKs mediate signal transduction activity *via* surface receptors for multiple cytokines (10, 11). These cytokines are integral to lymphocyte activation, proliferation, and function. Inhibiting the signaling pathways of these cytokines might result in the modulation of multiple aspects of the immune response (12). Tofacitinib may provide an effective treatment option in patients with an inadequate response to TNFi (13).

Difficult-to-treat RA, thus, blocking of additional pathways on top of the potential anti-inflammatory effect of TNF blockade appears a logical path forward (14). TNFi is one of the widely used bDMARDs in clinical practice. The population of RA patients who do not respond well to TNFi is large, and it is a challenge to further improve and approach a cure for this group of patients. They can choose other bDMARDs with different pathways, or tsDMARDs. Multi-drug failure is problematic in the management of D2T RA. As a result, some patients with multi-drug failure had a mean of 2.7 csDMARDs and 3.9 bDMARDs or JAKi, but treatment goal remained unachieved (15). Some patients resist all available treatment options agents and require new therapeutic target molecules.

TNFi and JAKi, are highly effective in the treatment of inflammatory arthritis, and are recommended by American College of Rheumatism and EULAR for the treatment of patients with moderate to severe RA. Tofacitinib, for example, mainly inhibits immune cell activation and the release of pro-inflammatory cytokines by selectively inhibiting the signaling pathways of JAK1 and JAK3, thereby directly or indirectly inhibiting multiple inflammatory factors including IL-6, TNF-α, and IL-1, which is different from the single-target inhibition of biologics (e.g., TNF, IL-6, IL-1, etc.) (2). In cases in which a biologic therapy is ineffective, the most common practice used by clinicians is to switch from a bDMARD to a different type of bDMARD or to a JAKi. The combinations of bDMARDs is not recommended for safety. JAKi and TNFi dual therapy is a potential treatment strategy for difficult-to-treat RA deserve to be discussed. Lydia Ntari et al. (16) investigated the effect of the Dasatinib and bosutinib, on the human TNF-dependent Tg197 arthritis mouse model. These data highlight the potential therapeutic advantage of an alternative therapeutic scheme involving the combination of a low dose TNFi with JAKi with potential clinical benefit for the treatment of arthritis and related comorbidities.

Dual agents targeting different inflammatory pathways is a potential treatment for severe and refractory diseases. An ongoing clinical trial which is listed at https://clinicaltrials.gov/ct2/show/NCT04870203. The investigators consider that there is a need for investigation into the addition of adalimumab to baricitinib in patients suffering of difficult-to-treat RA (inadequate response to TNFi). Baricitinib inhibits many pro-inflammatory cytokines involved in the pathogenesis of RA, but does not block the signal transduction downstream of TNF. Because of their interest in combining different mechanisms of action, the researchers plan to evaluate the efficacy and safety of a combination of baricitinib and TNFi (Adalimumab).

Dual therapy is used in the management of inflammatory bowel disease and was proven to be safe and efficacious (**Table 1**). Lee, SD et al. (17) identified 19 patients treated with Tofacitinib and biologic agents for refractory Crohn’s Disease between 2017 and 2019 and discovered that combining Tofacitinib with biologic agents were effective in achieving clinical and endoscopic improvement in some patients with severe, refractory Crohn’s Disease. Lee, JA et al. (18) presented a case of a patient with ulcerative colitis and seronegative inflammatory spondyloarthritis who achieved remission with the dual therapy of vedolizumab and Tofacitinib. Dolinger MT et al. (19) collected data from refractory pediatric inflammatory bowel disease patients receiving dual therapy. The data suggested that dual therapy may be considered in patients with limited therapeutic options. Even so, large-sample reports are yet to be published using the efficacy of combining bDMARDs with tsDMARDs in the treatment of other autoimmune disorders.

**Table 1:** Reported cases of Tofacitinib combined with Biologic Therapy.

Author	Year of Publication	Type of the Study	Diagnose	Description
Lee, S D et al.(17)	2022	Review	Refractory Crohn's Disease	Combination tofacitinib and a biologic was effective in achieving clinical and endoscopic improvement in refractory Crohn's Disease,which no new safety signals were observed.
Lee, J A et al.(18)	2020	Letter	Ulcerative Colitis and Seronegative Inflammatory Spondyloarthritis	A patient with Ulcerative Colitis and Seronegative Inflammatory Spondyloarthritis who achieved remission with combination vedolizumab and tofacitinib.
Dolinger, M T et al.(19)	2021	Original research	Refractory Pediatric Inflammatory Bowel Disease	Nine were treated with vedolizumab/tofacitinib, 4 with ustekinumab/vedolizumab, and 3 with ustekinumab/tofacitinib. Twelve achieved steroid-free remission at 6 months.One patient on 30 mg of vedolizumab/tofacitinib and prednisone daily developed septic arthritis and a deep vein thrombosis.

We describe a case of a 48-year-old woman with difficult-to-treat RA who tested positive for interferon gamma release assays (IGRAs) and was treated with Tofacitinib combined with adalimumab. Her condition remained in complete remission for more than one year of follow-up. Her laboratory tests and Disease Activity Score for 28-joint counts based on the erythrocyte sedimentation rate (DAS28-ESR), were all negative after accepting Tofacitinib and adalimumab at the same time. This case may be an excellent example for rheumatologists to consider combining tsDMARDs and bDMARDs as a new therapeutic strategy for difficult-to-treat RA.

## 2 Case description

A 48-year-old female patient diagnosed with RA in 2018 had been managed with methotrexate (MTX) monotherapy, MTX combined with prednisone, leflunomide (LEF), etanercept, or baricitinib. Unfortunately, satisfactory outcomes were not achieved in this patient. At the time of 2018, her IGRAs was negative and DAS28-ESR a high score, indicating that her disease was highly active. The patient presented to our clinic on November 16, 2020. She diagnosed with RA (**Table 2**). Her IGRAs was tested positive in 2022. Since the chest computed tomography (chest CT) did not suggest tuberculosis, rifampicin was used to prevent her from developing Latent tuberculosis infection (LTBI).

**Table 2:** Clinical information of the patient on November, 2020.

Laboratory text	Results	Normal range
C-reactive protein (CRP)	17	0-8mg/L
Erythrocyte sedimentation rate (ESR)	54	0-20mm/h
Rheumatic factor (RF)	51.8	0-20.0IU/ML
Anti-citrullinated protein antibody (Anti-CCP)	>200	0-5U/ML
Triglyceride	2.27	0.43-1.70mmol/L
Tuberculosis test (T-SPOT)	Positive	Negative
Hepatitis C antibody	Negative	Negative
Hepatitis B surface antigen (HBsAg)	Negative	Negative
Clinical Characteristics		
Tender joint count	20	
Swollen joint count	12	
Visual Analogue Scale(VAS)	90	
Smoking	No	
Drinking	No	
Heart disease	Deny	
Family history of RA	No	

On November 16, 2020, she started on MTX combined with Tofacitinib (Simcere Pharmaceutical Co. Ltd.), prednisone, and rifampin. Two months later, she had not improved. Duloxetine was added to her treatment regime to manage a suspected fibromyalgia syndrome, but the patient was still nonresponsive to it.

She has been using Tofacitinib in combination with MTX for more than 3 months from November 2020 to January 2021. However, the pain and fatigue tortured her. DAS28-ESR on January 14, 2021 (>5.1) showed that she was still in high active condition.

After in-depth communication with the patient, based on economic cost and convenience, we initiated a new treatment strategy on February 23, 2021, as MTX (10mg weekly) combined with Tofacitinib (5mg twice a day, Simcere Pharmaceutical Co. Ltd.), Adalimumab (40mg every two weeks, Simcere Pharmaceutical Co. Ltd.) and prednisone (10mg daily).

The patient had been on long-term irregular glucocorticosteroid therapy between 2018 and 2020. We used 15mg of prednisone at the beginning. The dose of prednisone was tapered from 15mg to 10mg and then 5mg daily. Finally, the prednisone was discontinued.

**Figure 1:**
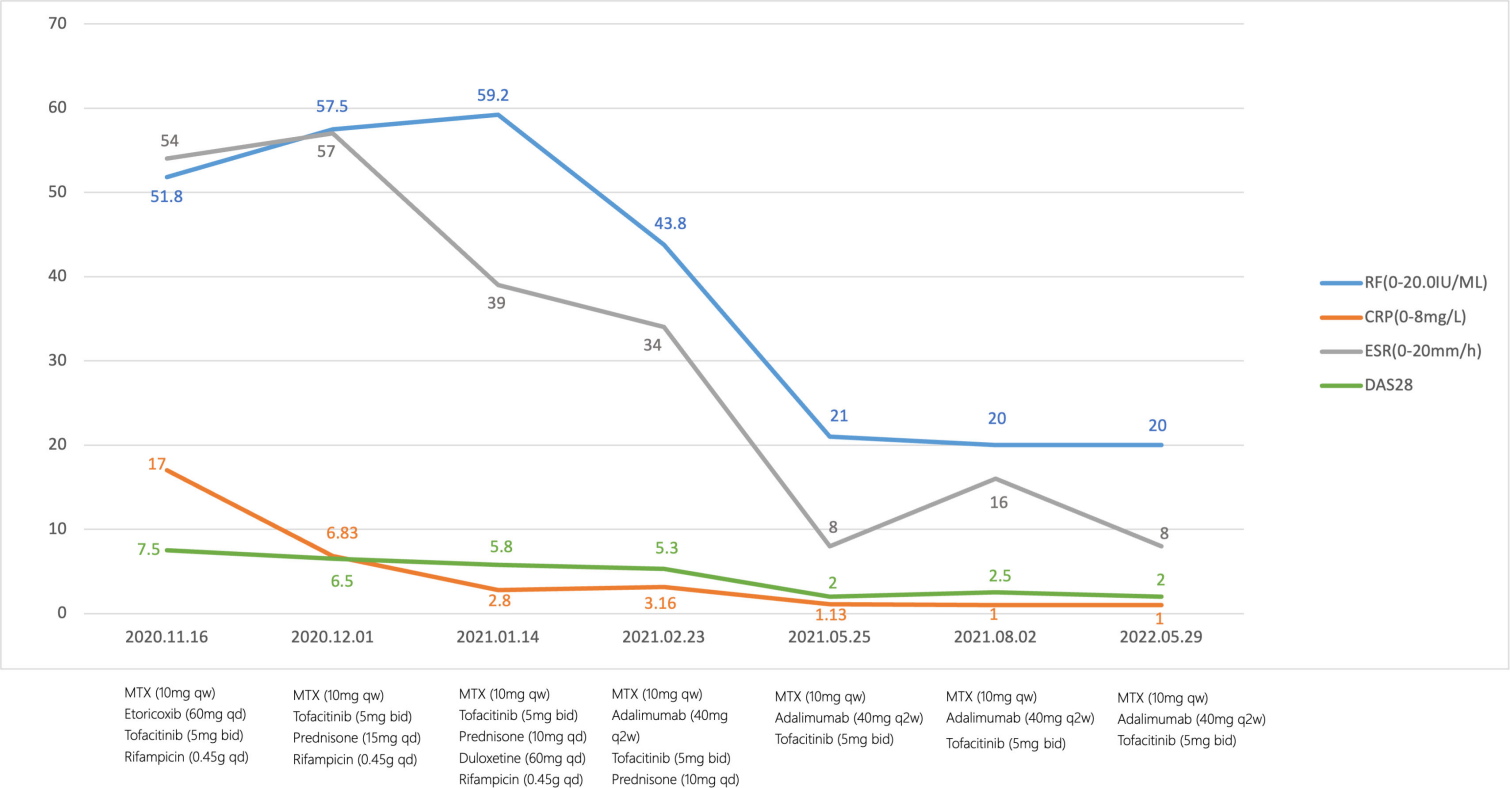
Flowchart.The therapeutic timeline of the patient. MTX, methotrexate; RF, rheumatoid factor; CRP, C-reactive protein; ESR, erythrocyte sedimentation rate; DAS28-ESR, Disease Activity Score for 28-joint counts.

This patient was followed for more than one year. DAS28-ESR was assessed routinely. We also monitored infection indicators, new-onset cardiovascular events, herpes zoster and other adverse events. Her condition was improved significantly, and she had no noticeable side effects. In the subsequent three months, prednisone was tapered and eventually stopped. In addition, her DAS28-ESR showed complete remission, for more than a year of follow-up. The patient did not develop LTBI, and she was also not diagnosed with herpes and thrombus (**Figure 1**).

## 3 Discussion

Concomitant syndromes or diseases such as fibromyalgia, osteoarthritis, and psychosocial factors associated with poor coping skills can result in non-inflammatory symptoms mimicking inflammatory activity (3). These non-inflammatory symptoms contribute to difficult-to-treat RA. A previous study also revealed that patients with RA had a more significant number of comorbidities and were less likely to achieve a therapeutic response (20). In our case, we added duloxetine because fibromyalgia syndrome was suspected; however, the patient still did not improve. In early treatment, rifampicin was used to prevent LTBI. It is worth noting that is a strong inducer of P450 isoenzymes. Rifampicin could decrease the concentration of Tofacitinib (21). This may affect the effectiveness of the treatment. However, her fatigue, joint swelling and pain continued to be relieved after discontinuation of rifampicin.

For the primary failure of a drug, switching to a drug with another mechanism of action is suggested. For secondary failure, switching to a drug with a different and the same mechanism of action is suggested (2). Tofacitinib with adalimumab for 3 months. Unfortunately, she complained of recurrent joint swelling and pain. Given that she had previously used TNFi (etanercept) and JAKi (baricitinib) in 2018.She had already used multiple b/tsDMARDs but was ineffective, accompanied by significant financial stress. Of all the b/tsDMARDs therapy offered, she ultimately chose MTX combined with Tofacitinib, with poor effect.Finally, we initiated MTX combined with Tofacitinib, and adalimumab. Patients maintain dual therapy until now. Before using the combination therapy with adalimumab and tofacitinib, she had used etanercept, which was not effective. Due to financial cost and indications, the patient refused to use abatacept, tocilizumab, Rituximab, etc. Finally, we chose adalimumab combined with tofacitinib which was financially acceptable to her.

There are rarely reports or data on JAKi in combination with TNFi. But dual biologic therapy on Crohn’s Disease and ulcerative colitis suggests a new strategy on difficult-to-treat RA. Infection is the adverse event that we are specifically concerned about. In this case, the patient tested positive in IGRAs. She was treated with rifampin for three months and She was not diagnosed with tuberculosis for over a year of follow-up.

Tofacitinib may provide an effective treatment option in patients with an inadequate response to TNFi (13). This case report showed therapeutic improvement in RA patients who had poor results with TNFi, however, in combination with JAKi. During MTX combined with adalimumab, MTX combined with Tofacitinib, patient had high level of inflammatory tests (C-reactive protein and Erythrocyte sedimentation rate), accompanied by severe painful joint swelling. However, MTX combined with tofacinitib or adalimumab has shown improved outcomes over monotherapy with any of those drugs. Significant increase in pro-inflammatory cytokines such as TNF-α, IL-1ß, IL-6 and IFN-γ demonstrates that cytokines can accelerate the progression of RA. Some of these cytokines affect RA mainly through the JAK/STAT pathway. Although the TNF receptor does not directly signal through JAKs, synoviocyte activation by TNF stimulation can be affected by JAK-dependent pathways. TNF can activate JAK/STAT pathway by causing STAT3 phosphorylation (22). We speculate that in JAKi and TNFi dual therapy, the inflammatory pathway in difficult-to-treat RA patients is more fully inhibited, thus improving the remission. Targeting multiple inflammatory cytokines in combination may lead to more effective treatment and enhanced clinical responses in difficult-to-treat RA patients compared to the current second-line treatment strategies. The safety of dual therapy is a matter of concern. There were no new infections in our patients during the follow-up of more than one year.

## 4 Conclusion

This case report, describes a satisfactory outcome in a difficult-to-treat RA patient treated with Tofacitinib combined with adalimumab. JAKi and TNFi dual therapy could be a potential treatment strategy for difficult-to-treat rheumatoid arthritis without inducing infectious diseases, such as tuberculosis. However, there is much room for discussion regarding the safety and efficacy of dual therapy.

## 5 Limitation

This is a case report, only one patient was studied. A large sample size clinical study is needed to support this combination strategy in the future. Besides, the patient had used two biological agents which were ineffective. However, those bDMARDs were all TNFi inhibitors. She did not use other bDMARDs with different mechanisms, such as IL-6 inhibitors, CTLA4 inhibitors, which would be effective to her.

## Data availability statement

The original contributions presented in the study are included in the article/supplementary material. Further inquiries can be directed to the corresponding author.

## Ethics statement

The experimental protocol was established, according to the ethical guidelines of the Helsinki Declaration and was approved by the Human Ethics Committee of The First Affiliated Hospital of Guangzhou University of Chinese Medicine(NO.K-2022-084). No identifying information was included in the study. Written informed consent was obtained from the individual for the publication of any potentially identifiable images or data included in this article.

## Author contributions

J-WC and W-SZ contributed to data collection and drafting of the manuscript. QX and C-SL helped revise the manuscript. All authors contributed to the article and approved the submitted version.
